# Supplementary material for: A metal-supported single-atom catalytic site enables carbon dioxide hydrogenation
Source: Nat Commun. 2022 Feb 10;13:819. doi: 10.1038/s41467-022-28456-9 (PMC8831533; doi:10.1038/s41467-022-28456-9)
Supplement: Supplementary file 1 — Supplementary Information [file 41467_2022_28456_MOESM1_ESM.pdf]

SUPPLEMENTARY INFORMATION

A Metal-Supported Single-Atom Catalytic Site Enables Carbon Dioxide Hydrogenation

Sung-Fu Hung[1,2,4], Aoni Xu[1,4], Xue Wang[1,4], Fengwang Li[1,4], Shao-Hui Hsu[3], Yuhang Li[1], Joshua Wicks[1], Eduardo González Cervantes[1], Armin Sedighian Rasouli[1], Yuguang C. Li[1], Mingchuan Luo[1], Dae-Hyun Nam[1], Ning Wang[1], Tao Peng[1], Yu Yan[1], Geonhui Lee[1], Edward H. Sargent[1,*]

[1]Department of Electrical and Computer Engineering, University of Toronto, Toronto, ON, Canada.

[2]Department of Applied Chemistry, National Yang Ming Chiao Tung University, Hsinchu 300, Taiwan.

[3]Taiwan Semiconductor Research Institute, National Applied Research Laboratories, Hsinchu 300, Taiwan.

[4]These authors contributed equally

Email: ted.sargent@utoronto.ca

This file includes:

Supplementary Note 1 and 2
Supplementary Figures 1 to 41
Supplementary Tables 1 and 2
References

Supplementary Note 1

We still detect a small amount of ethylene (2%) in Cu-FeSA and attribute this to the C-C coupling on Cu atoms, far from Fe atoms, that still adsorbs the CO intermediates. In order to evaluate this contribution, we calculate the atomic ratio of Cu and Fe at the surface via the results of electrochemical active surface area and ICP-mass in Supplementary Fig. 17 (Cu:Fe = 14:1, showing below). We find that the uniform atomic arrangement in this atomic ratio (Supplementary Fig. 18) can maximize the migration of the CO intermediates to enhance the methane production and suppress the ethylene production. The minor amount of ethylene should come from the non-ideal atomic arrangement of Cu that performs the C-C coupling.

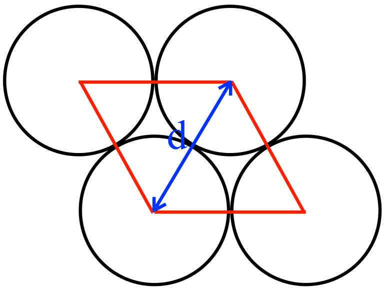

To calculate the quantity of Cu/Fe atoms at the surface of Cu-FeSA, we utilize ICP-mass spectrometry to obtain the atomic amount of Fe while we measure the electrochemical active surface area (ECSA) to evaluate the atomic amount of Cu.

(1) With a 0.3 at% of Fe in Cu-FeSA as determined via ICP-mass spectrometry, the surface density of Fe atoms is 1.13×10^{16} #/cm^2.

(2) Assuming the Cu (111) arrangement at the surface,

The area for 1 Cu: $2 \times d/2 \times \sqrt{3}d/2 = \sqrt{3}d^2/2 = 1.42 \times 10^{-16}$ cm^2 ($d_{Cu} = 1.28$ Å)

The double layer capacitance (C_{dl}) for Cu-FeSA is 670 µF, as shown in Supplementary Fig. S16. We divide this value by the specific C_{dl} (29 µF) of a smooth copper to obtain an ECSA of 22.4 cm^2. Thus, the amount of Cu atoms at the surface is 1.58×10^{17} #/cm^2 ($22.4 / 1.42 \times 10^{-16}$ cm^2)

(3) Cu:Fe = $1.58 \times 10^{17} : 1.13 \times 10^{16} \approx 14:1$

We further calculate the turnover frequency (TOF) for the bare Cu and Cu-FeSA.

TOF = Turnover number for methane formation (TON) / Number of metal atom (N).

TON = (Electron x Faradaic efficiency) / (Electron for methane production x Faraday constant)

N = (Metal concentration by ICP x Avogadro number) / Molecular weight of metal

For bare Cu,

TON = (0.2 x 3600 x 2.08% x 6.02 x 10^{23}) / (8 x 96485.3)

N = 294.208 x 10^{-6} x 6.02 x 10^{23}) / 63.546

TOF = TON / N = 4.19 h^{-1}

For Cu-FeSA,

TON = (0.2 x 3600 x 64% x 6.02 x 10^{23}) / (8 x 96485.3)

N = 1.045 x 10^{-6} x 6.02 x 10^{23}) / 55.845

TOF = TON / N = 31770 h^{-1}

Supplementary Note 2

Reaction free energy calculation

The product selectivity of CO_2 reduction is strongly dependent on two competing reactions starting from *CO intermediate,

*CO protonation for CH_4 production: *CO + H2O + e- → *COH + OH^-

or *CO + H2O + e- → *CHO + OH^-

C–C coupling: *CO + *CO + H_2O + e- → *OCCOH + OH-

where the * represent the adsorption site.

The hydrogenation of CO on different catalysts surfaces for methane production were simulated according to the following reaction through (1) the *COH reaction pathway and (2) the *CHO reaction pathway,

(1) *COH: *CO + H_2O → *COH + OH^-

*COH + H_2O + e^- → *C + OH^-

*C + H_2O → *CH + OH^-

*CH + H_2O → *CH_2 + OH^-

*CH_2 + H_2O → *CH_3 + OH^-

*CH_3 + H_2O → *CH_4 + OH^-

(2) *CHO: *CO + H_2O → *CHO + OH^-

*CHO + H_2O → *CHOH + OH^-

*CHOH + H_2O + e^- → *CH + OH^-

*CH + H_2O → *CH_2 + OH^-

*CH_2 + H_2O → *CH_3 + OH^-

*CH_3 + H_2O → *CH_4 + OH^-

The reaction energies for these were obtained by

$$\Delta G = \Delta E^{DFT} + \Delta ZPE - T\Delta S$$

where ΔE^{DFT} is the reaction energy calculated from DFT; ΔZPE is zero-point energy, which was neglected here; ΔS is the change in entropy, whose values of gaseous molecules are taken from the standard database in the NIST web-book.[1]

Taking the CO hydrogenation reaction *CO + H_2O + e^- → *COH + OH^- as example, the reaction energy is equals to

$$\Delta G = G(*COH) – G(*CO) + G(OH^-) - G(H_2O) - G(e^-)$$

where, G(*COH) and G(*CO) is the total free energy of *COH and *CO adsorption configuration; G(OH^-) is the free energy of hydroxyl ion, to calculate this value, we assume the equilibrium

$$H_2O \leftrightarrow H^+ + OH^-$$

Which relates the chemical potentials as

$$\mu_{H^+} + \mu_{OH^-} = \mu_{H_2O}$$

Then,

$$\mu_{H^+} + \mu_{e^-} + \mu_{OH^-} - \mu_{e^-} = \mu_{H_2O}$$

Thus,

$$\mu_{OH^-} - \mu_{e^-} - \mu_{H_2O} = -(\mu_{H^+} + \mu_{e^-})$$

Here, $\mu_{H^+} + \mu_{e^-}$ can be calculated using computational hydrogen electrode (CHE) model developed by Nørskov and co-workers.[2,3]

Supplementary Figures

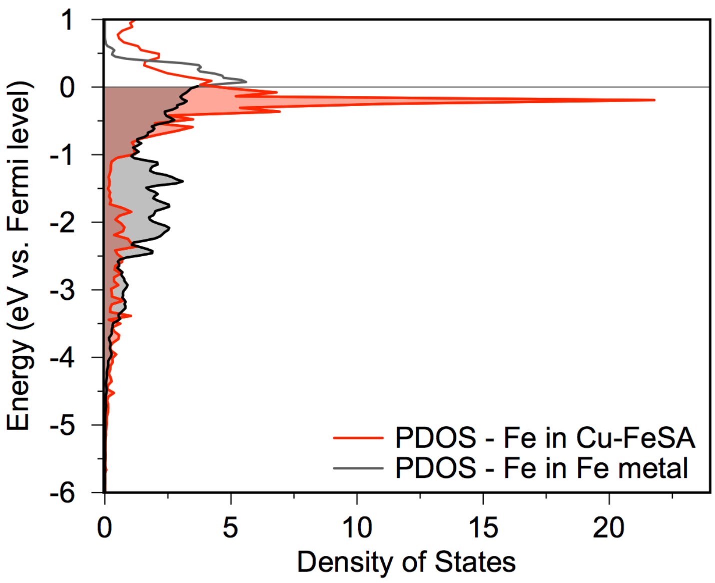

Supplementary Fig. 1 | Comparison of density of states of Fe *d* orbitals between the Cu-supported single-atom iron with the metallic iron

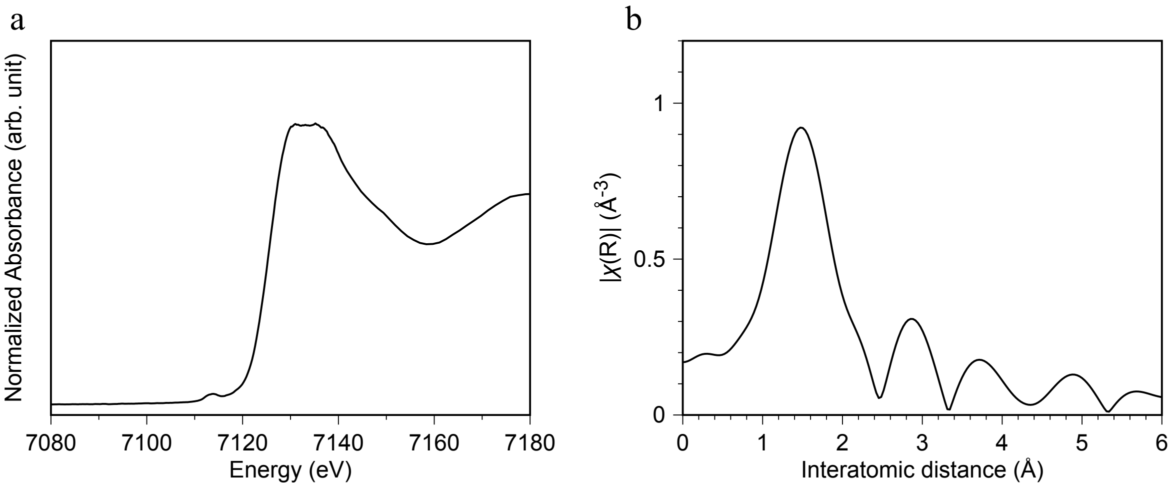

Supplementary Fig. 2 | Fe K-edge X-ray absorption spectroscopy of Fe cluster on the copper surface. (a) X-ray absorption near-edge structure (XANES). (b) Extended X-ray absorption fine structure (EXAFS).

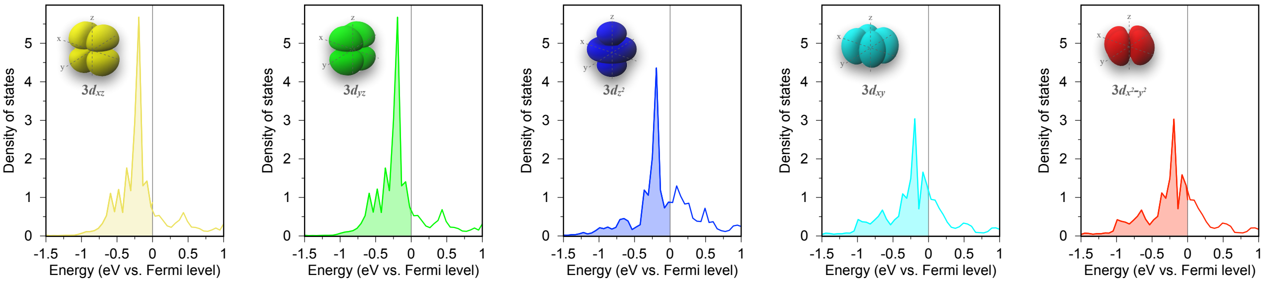

Supplementary Fig. 3 | Individual *d* orbitals of Fe in Cu-FeSA.

Catalysts	Adsorption sites	Initial configurations	Optimized configurations	ΔE (eV)
Cu	top	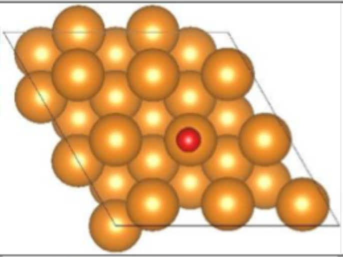	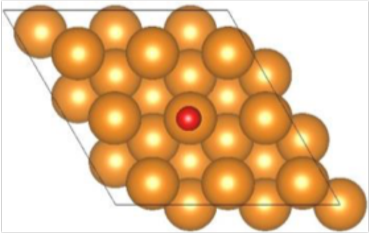	-0.69
	bridge	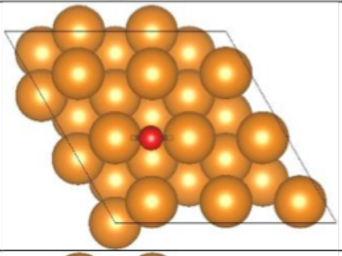	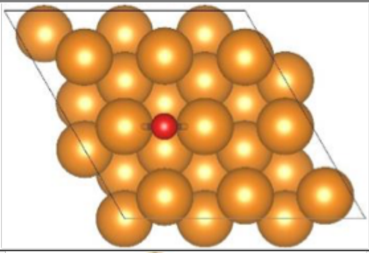	-0.73
	fcc	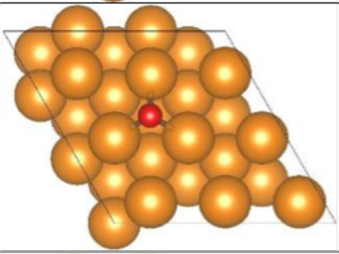	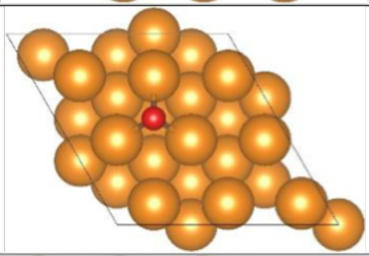	-0.80
	hcp	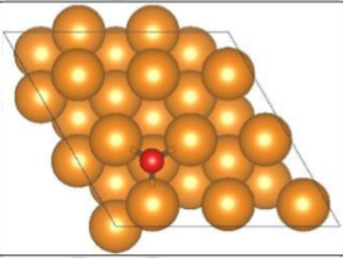	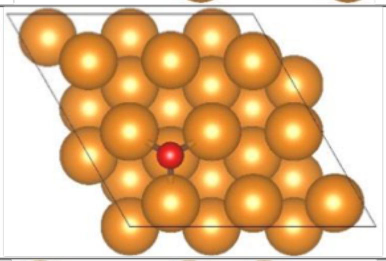	-0.79
CuFe SAA	top-Fe	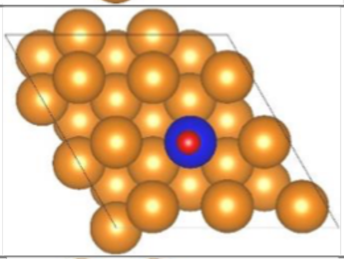	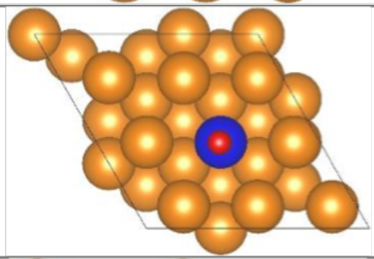	-2.50
	bridge-Fe	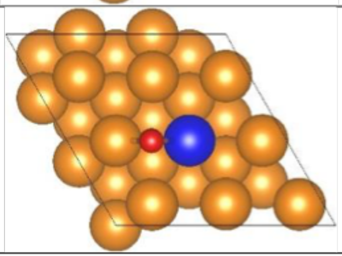	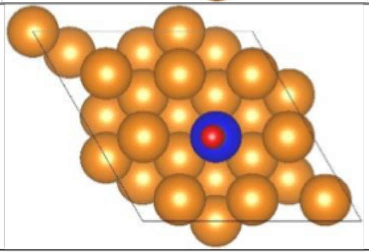	-2.50

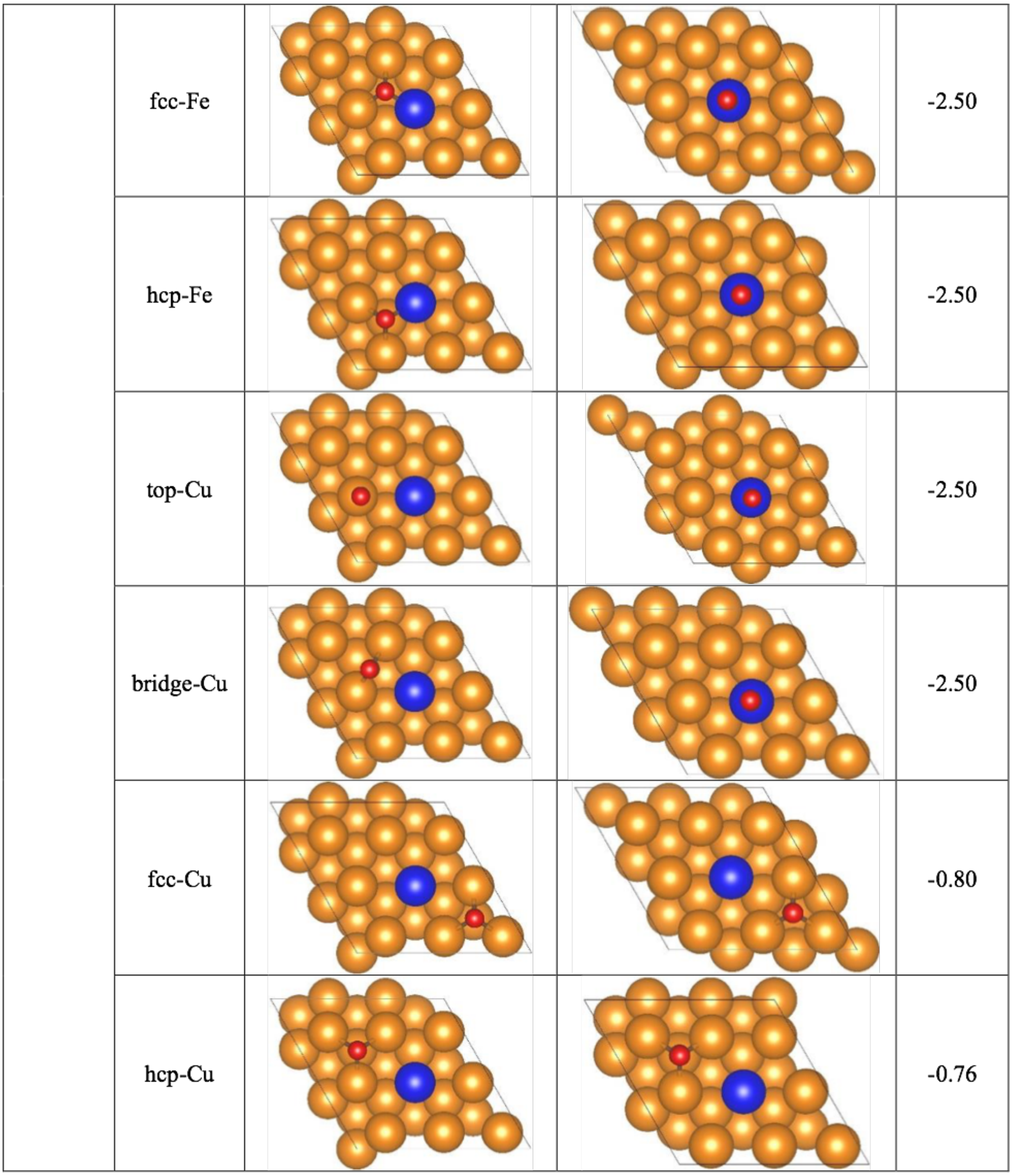

Supplementary Fig. 4 | CO intermediates adsorbed on various surface positions before and after optimization in pristine Cu and Cu-FeSA. In comparison with the initial and optimized configurations, we found that CO intermediates adsorbed to a neighboring atom migrate to the atop site of the iron atom.

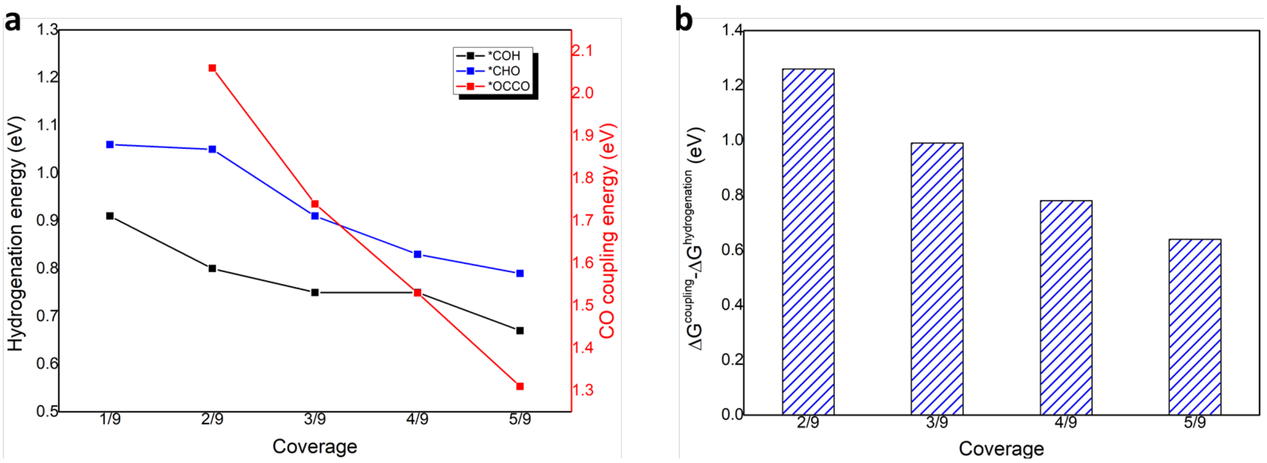

Supplementary Fig. 5 | Coverage effect. (a) reaction energy for CO hydrogenation (*COH and *CHO) and CO coupling steps on Cu-FeSA at different CO coverage; both hydrogenation and coupling reactions are promoted with increasing CO coverage (b) preference of CO coupling (defined as reaction energy difference between coupling step and hydrogenation step) at different CO coverage. With CO coverage increases from 2/9 (2 *CO on surface with 9 metal atoms) to 5/9, the reaction energy of CO coupling gradually decreases and close to hydrogenation energy, which suggests an improved priority of C_2 products at high coverage.

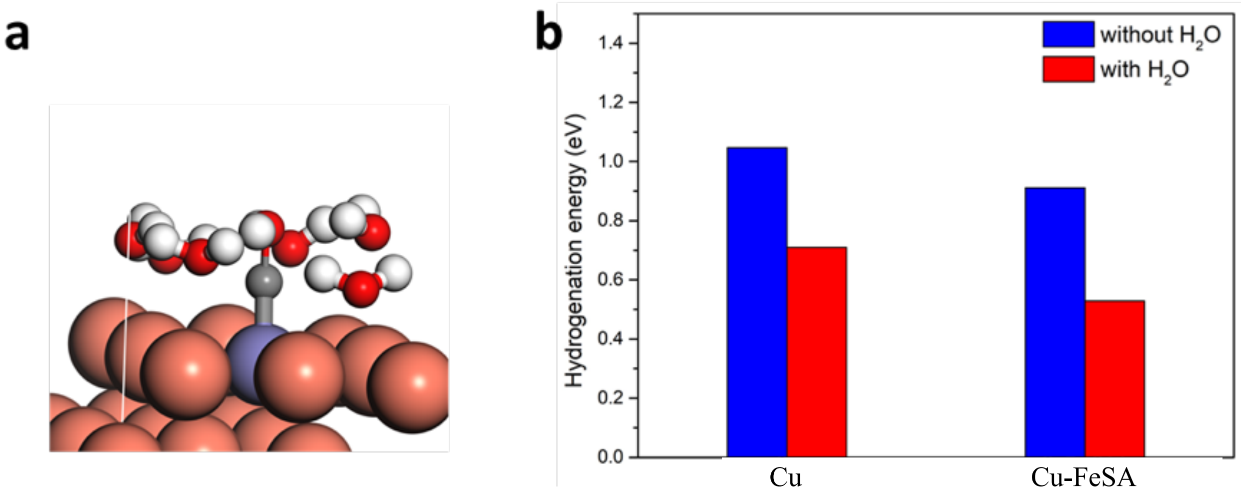

Supplementary Fig. 6 | Solvation effect on the CO hydrogenation through *COH pathway. (a) configuration of *COH adsorbed on Cu-FeSA with 6 H_2O molecules in model; (b) hydrogenation energy results of Cu and Cu-FeSA with and without solvation effect. Solvent decreases the hydrogenation energy on both Cu and Cu-FeSA in the pathways of *COH, but it does not change the catalytic mechanism and behavior.

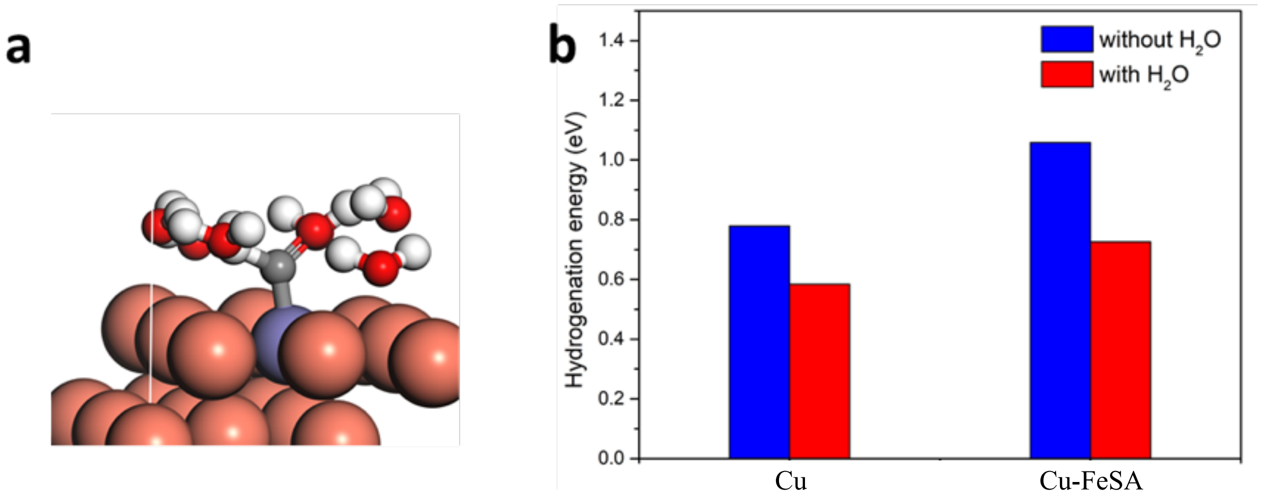

Supplementary Fig. 7 | Solvation effect on the CO hydrogenation through *CHO pathway. (a) configuration of *CHO adsorbed on Cu-FeSA with 6 H_2O molecules in model; (b) hydrogenation energy results of Cu and Cu-FeSA with and without solvation effect. Solvent decreases the hydrogenation energy on both Cu and Cu-FeSA in the pathways of *CHO, but it does not change the catalytic mechanism and behavior.

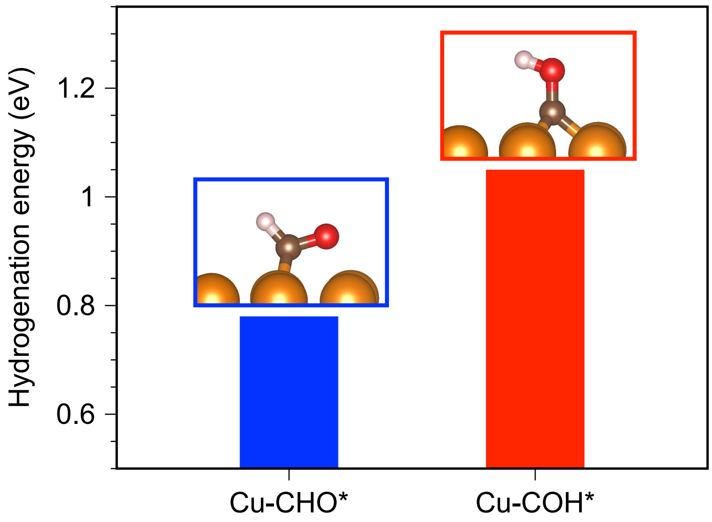

Supplementary Fig. 8 | Hydrogenation energy of the intermediates for methane production on the pristine Cu. Even though pure Cu has a lower (~0.3 eV) hydrogenation energy through *CHO reaction pathway, an energy difference ~1 eV of CO coupling between pure Cu and CuFe suggests a higher preference for C2 products on pure Cu compared to CuFe SAA.

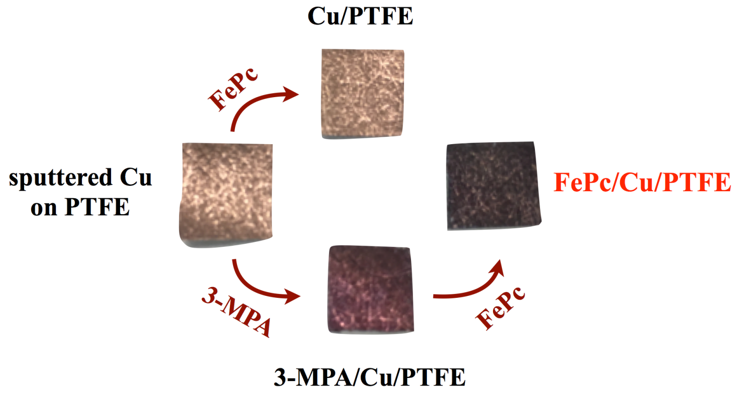

Supplementary Fig. 9 | Digital images of the gas diffusion electrodes before and after surface modification. FePc cannot directly be adsorbed on copper surface without 3-MPA modification, leading to the lack of visible change on the copper surface. The thiol end anchored copper displays the obvious color change to maroon. The self-assembly of FePc further changes the color to a dark purple.

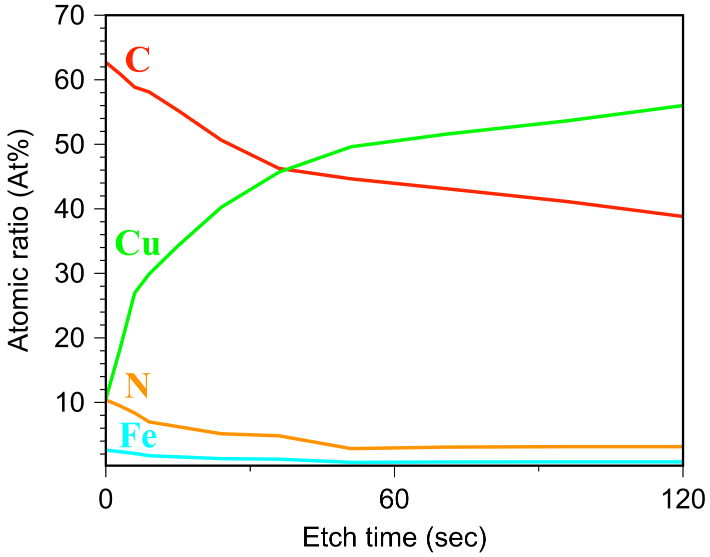

Supplementary Fig. 10 | Depth-profiling X-ray photoelectric photoelectron spectroscopy of iron-phthalocyanine-modified copper. The depth profile evidences iron ions near the copper surface.

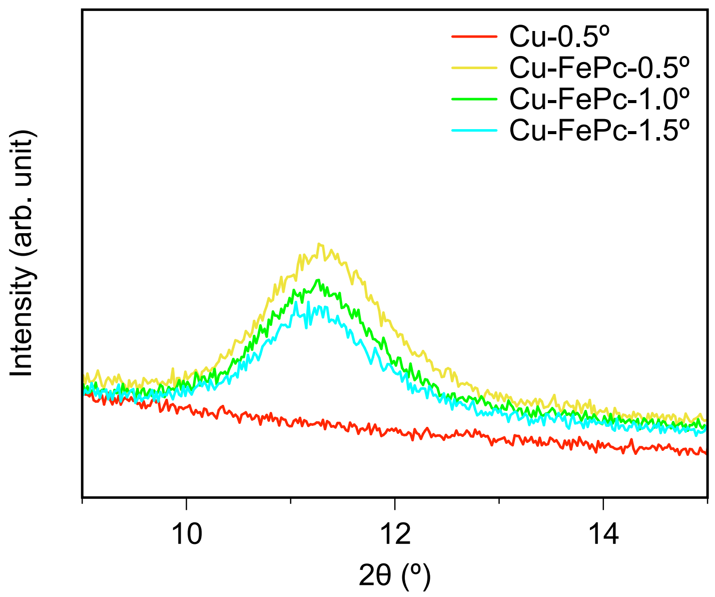

Supplementary Fig. 11 | Grazing-incidence-angle X-ray diffraction patterns with various grazing incident angles of pristine and iron-phthalocyanine-modified copper on flat Si wafer. No peak is observed at θ ~ 11.3° for pristine copper, indicating that the self-assembling iron phthalocyanine contributes the XRD signal. The lower intensity at higher grazing incident angle means that the peak should originate near the surface and the distance between the iron-phthalocyanine and the copper surface. We associate the XRD signal from this thin layer with adsorption at different facets for iron phthalocyanine on the rough surface of sputtered copper.

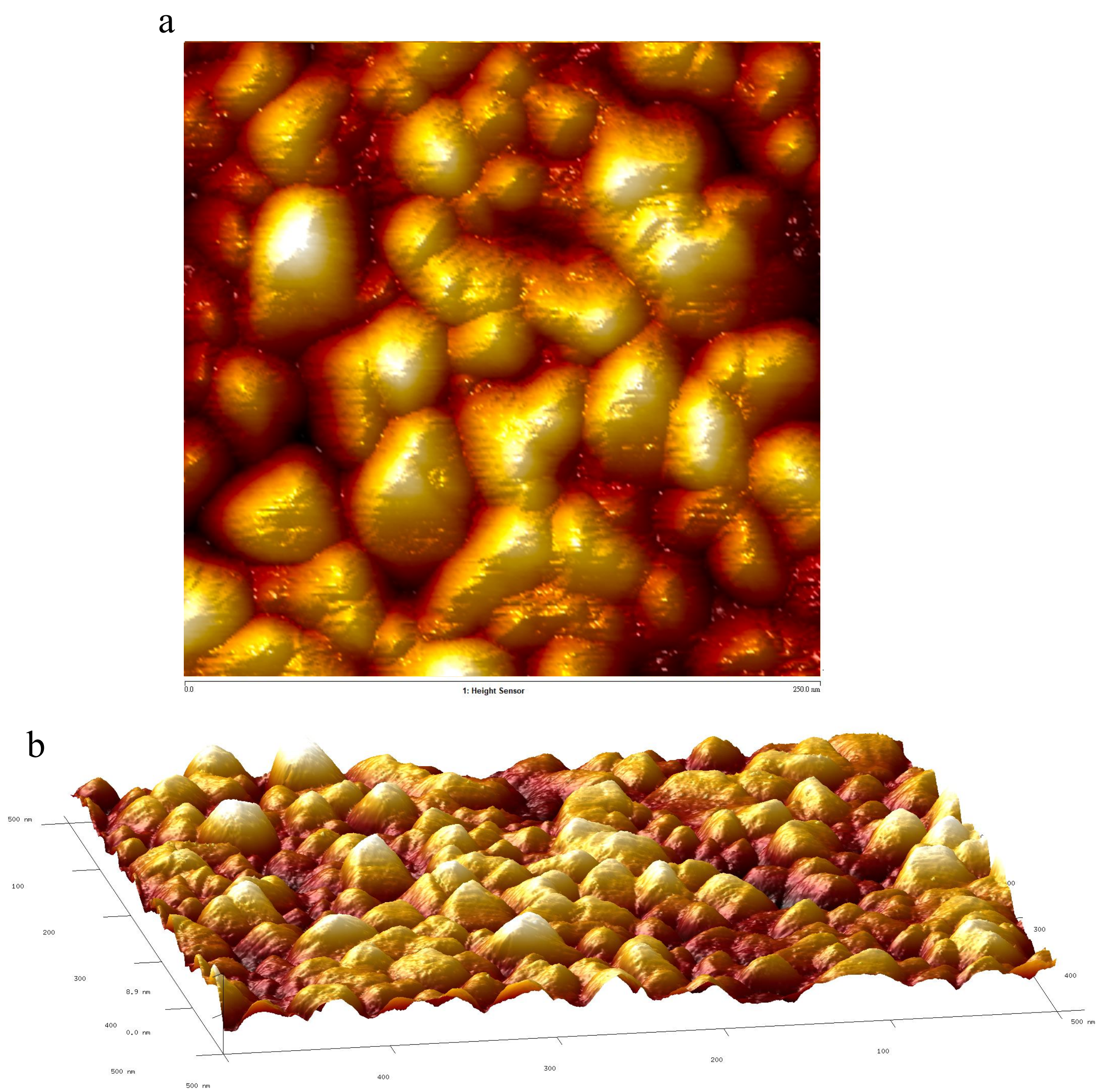

Supplementary Fig. 12 | Atomic force microscope images of sputtered copper on flat Si wafer.

(a) The scanning surface and (b) the 3D image of sputtered copper on flat Si wafer

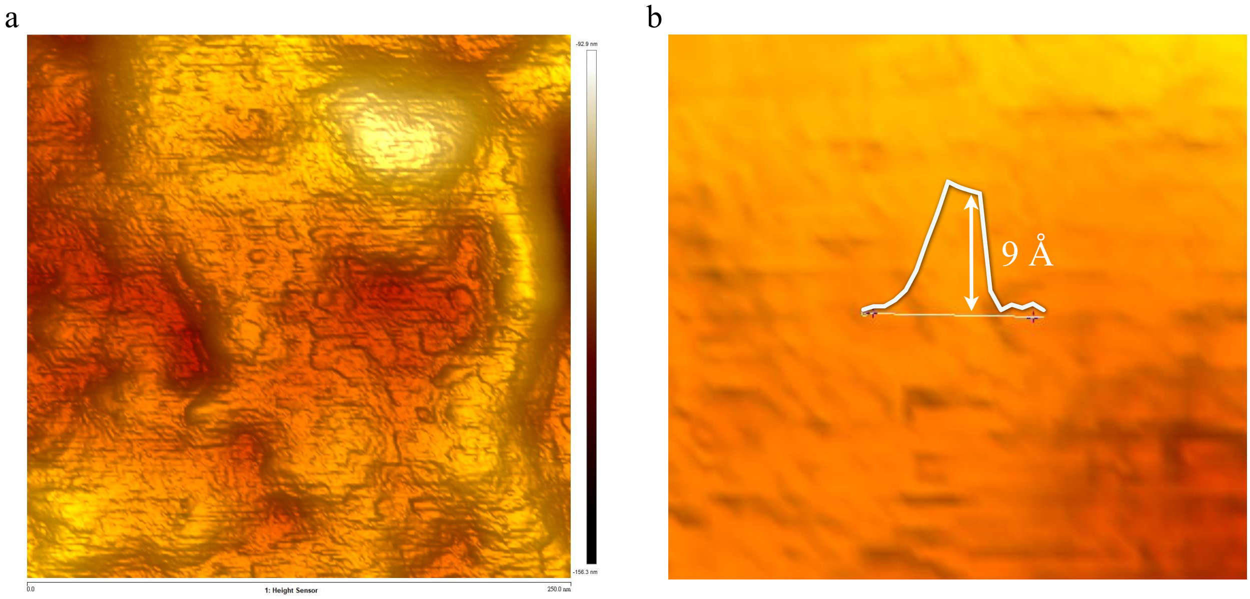

Supplementary Fig. 13 | Atomic force microscope images of iron-phthalocyanine-modified copper on flat Si wafer. (a) The scanning surface of iron-phthalocyanine-modified copper on flat Si wafer, suggesting that the dense and flat occupation of iron-phthalocyanine on copper surface. (b) The step analysis at the noncontinuous site.

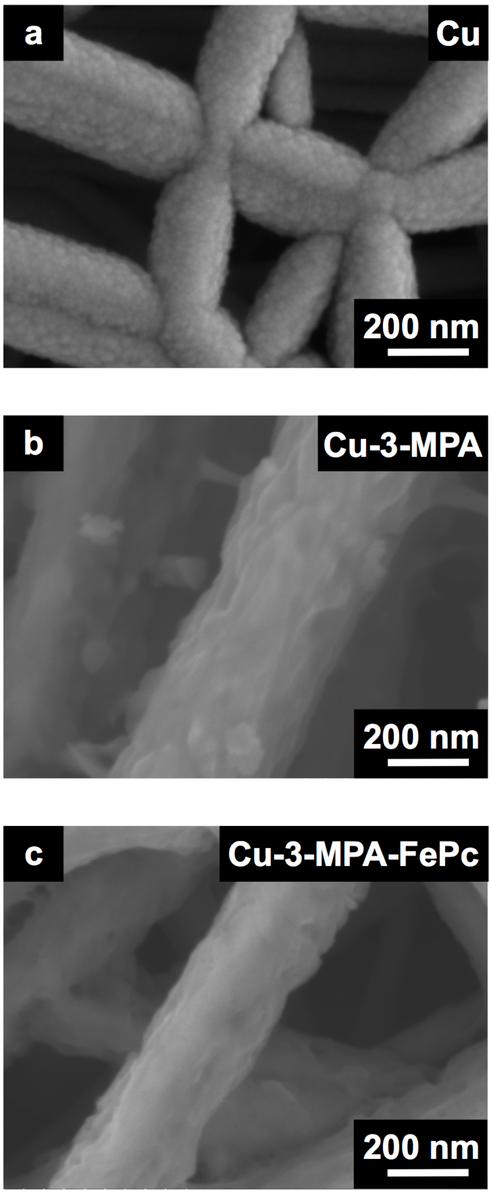

Supplementary Fig. 14 | Microstructural images of gas diffusion electrodes before and after surface modification. (**a**) Cu, (**b**) Cu-3-MPA, and (**c**) Cu-3-MPA-FePc.

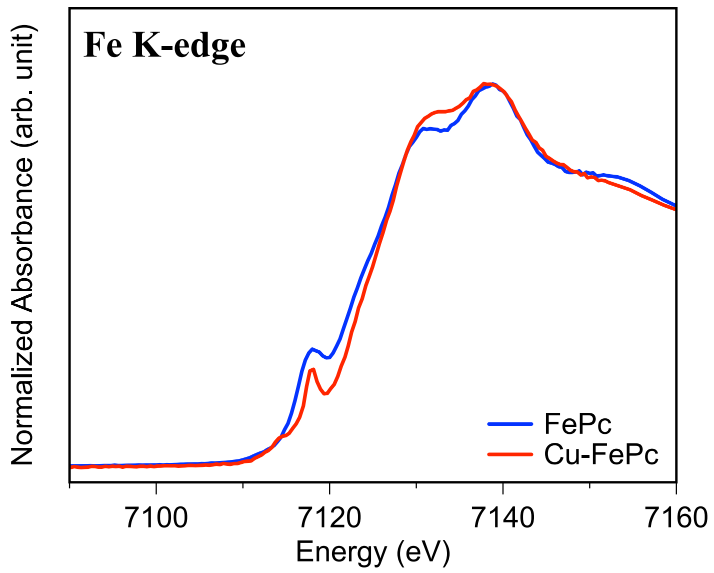

Supplementary Fig. 15 | Fe K-edge XANES spectra of iron-phthalocyanine-modified copper.

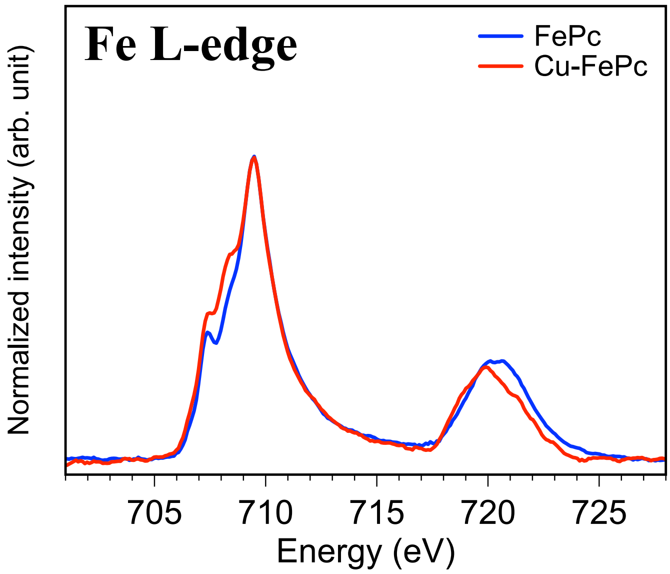

Supplementary Fig. 16 | Fe L-edge XAS spectra of iron-phthalocyanine-modified copper.

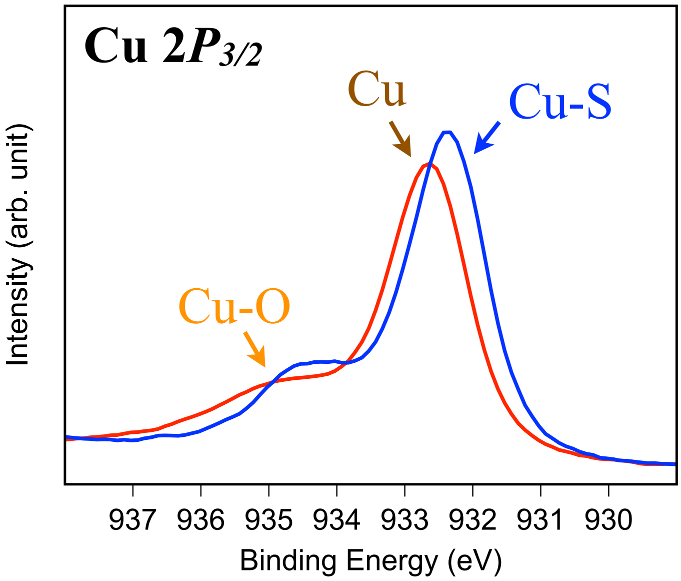

Supplementary Fig. 17 | X-ray photoelectron spectroscopy of Cu 2*P*$_{3/2}$ for iron-phthalocyanine-modified copper.

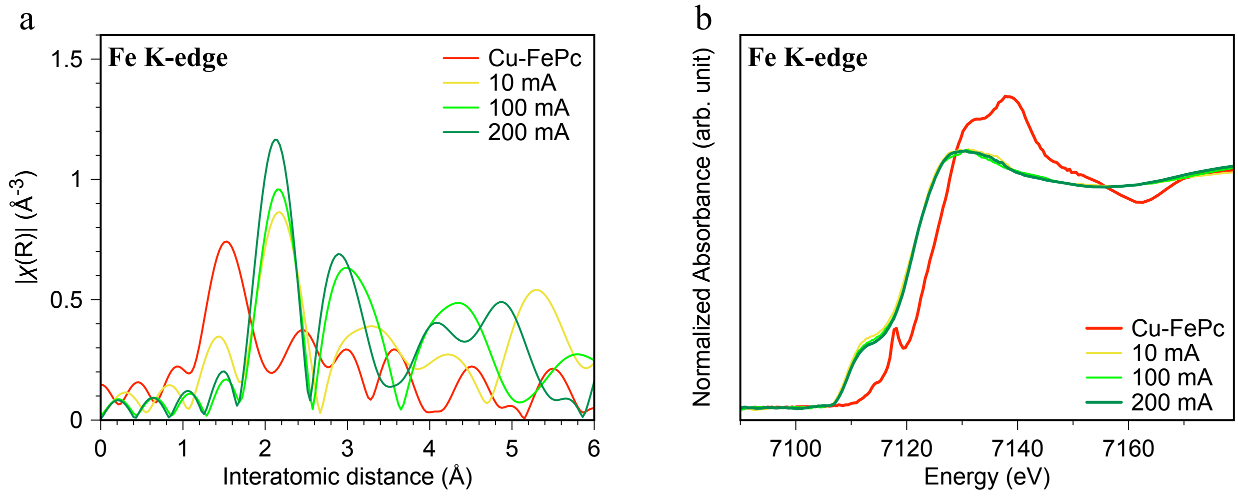

Supplementary Fig. 18 | *In-situ* X-ray absorption spectroscopy of Fe K-edge operating in flow cell at various current. (**a**) *In-situ* extended X-ray absorption fine structure (EXAFS) of Fe K-edge. (**b**) *In-situ* X-ray absorption near-edge structure (XANES) of Fe K-edge.

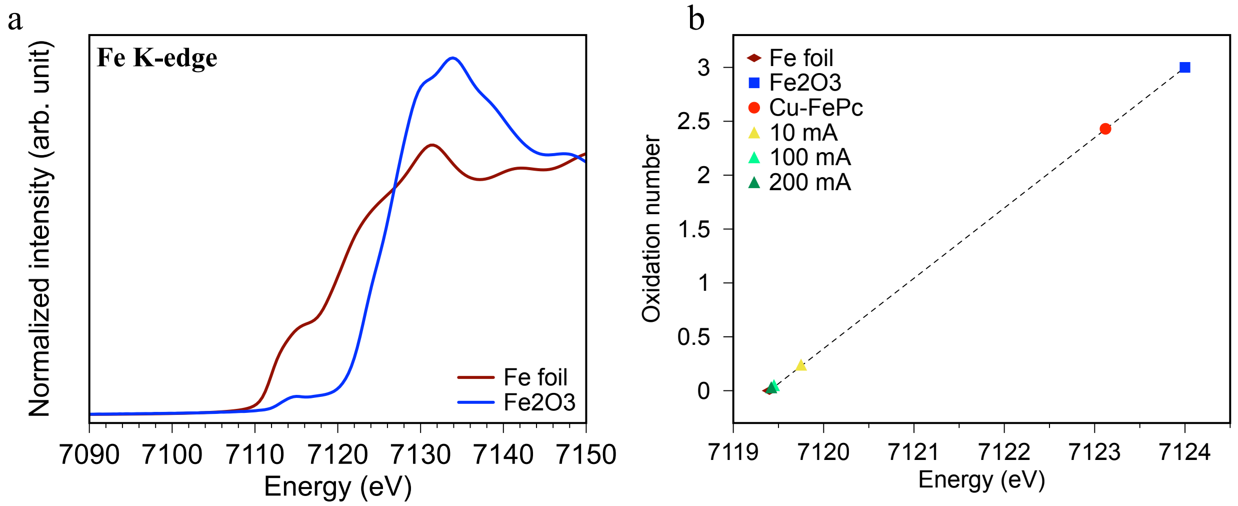

Supplementary Fig. 19 | Identification of oxidation states of Fe operating in flow cell at various current. (**a**) X-ray absorption near edge spectroscopy of standard samples. (**b**) Oxidation states of Fe in flow cell at various current. We use energy of the 0.5 of normalized intensity as the energy position for the oxidation states rather than the inflection point due to the pre-edge feature in the spectrum.

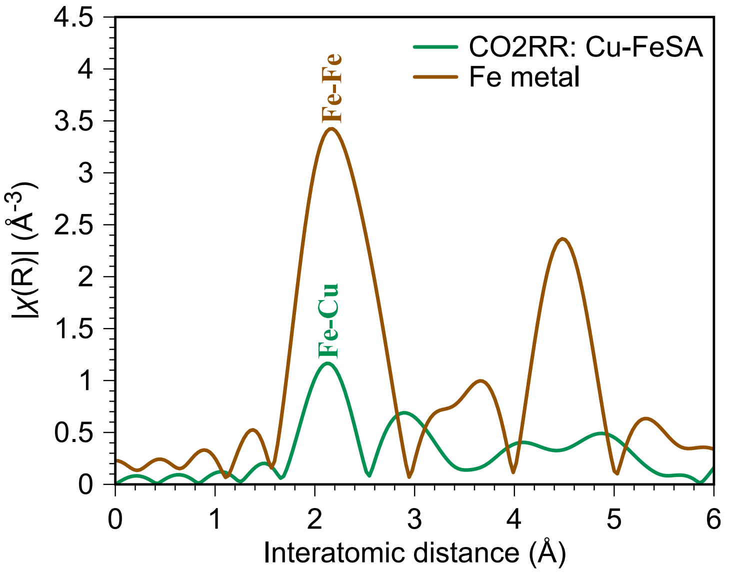

Supplementary Fig. 20 | EXAFS comparison of the Cu-supported single-atom iron with bulk metallic iron.

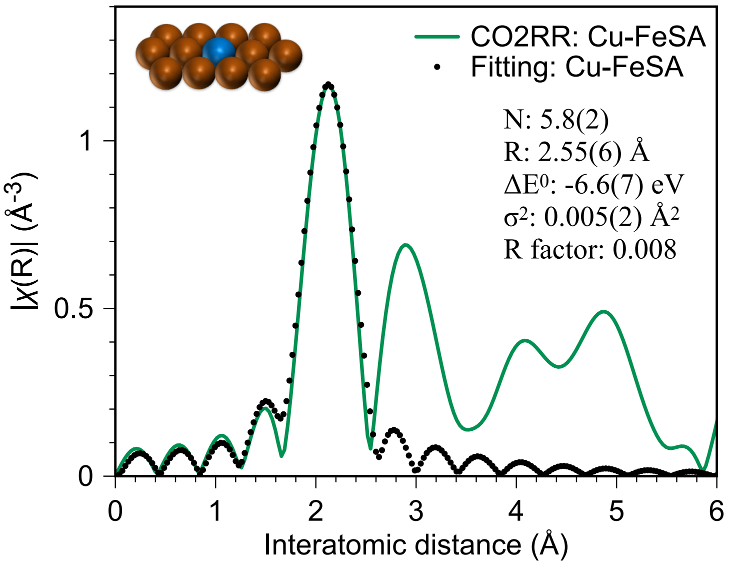

Supplementary Fig. 21 | Fitting of k^2 weighted Fe K-edge EXAFS for Cu-FeSA at the catalytic current of 200 mA. The k-range and R-range for the k^2-weighted Fe K-edge EXAFS are 3-10^{-1} Å and 1.6-2.5 Å, respectively. Fe-Cu path, obtained from the replacement of a Cu atom with a Fe atom in FCC structure (the inset shows the closest packed facet for iron occupation), was adopted for structural fitting. Fitted structural parameters from k^2 weighted EXAFS oscillations were also shown in this figure. N: coordination number; R: interatomic distance; σ^2: the EXAFS Debye−Waller factor. The data were processed through Athena software from the IFEFFIT package for analyzing XANES and EXAFS. REX2000 software using ab initio-calculated phases and amplitudes from the program FEFF 8.2 was used for EXAFS fitting.

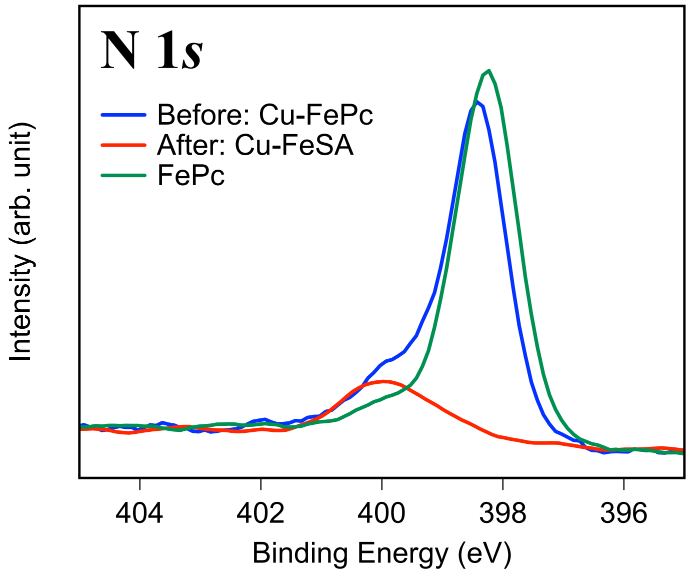

Supplementary Fig. 22 | *Ex-situ* XPS analysis of N 1*s* before and after catalytic CO_2 reduction reaction.

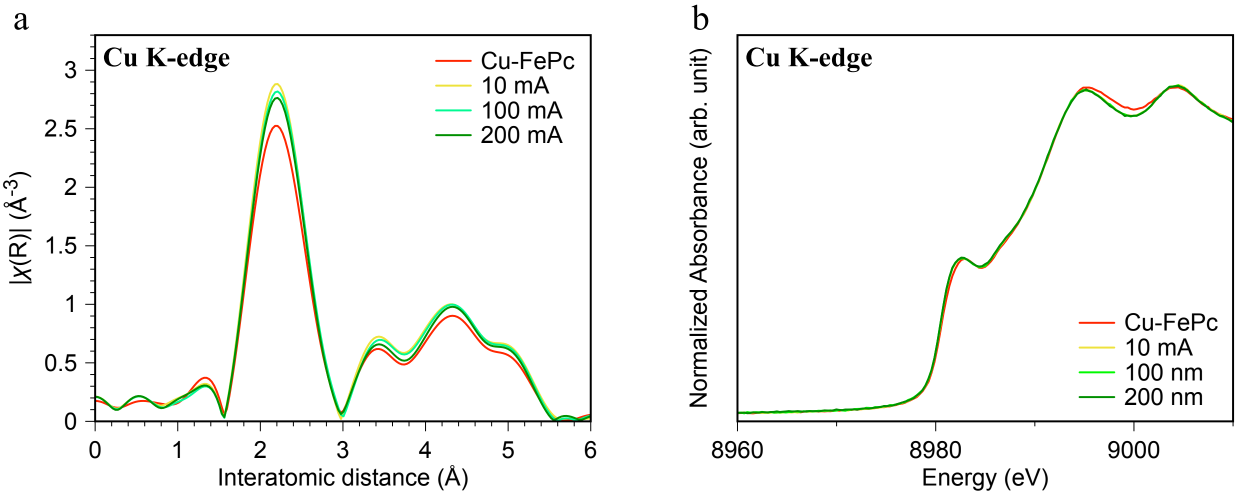

Supplementary Fig. 23 | In-situ X-ray absorption spectroscopy of Cu K-edge operating in flow cell at various current. (**a**) In-situ extended X-ray absorption fine structure (EXAFS) of Cu K-edge, showing a single Cu-Cu metallic bond without Cu-O bond. (**b**) In-situ X-ray absorption near-edge structure (XANES) of Cu K-edge. XANES suggests the oscillation behavior of metallic Cu.

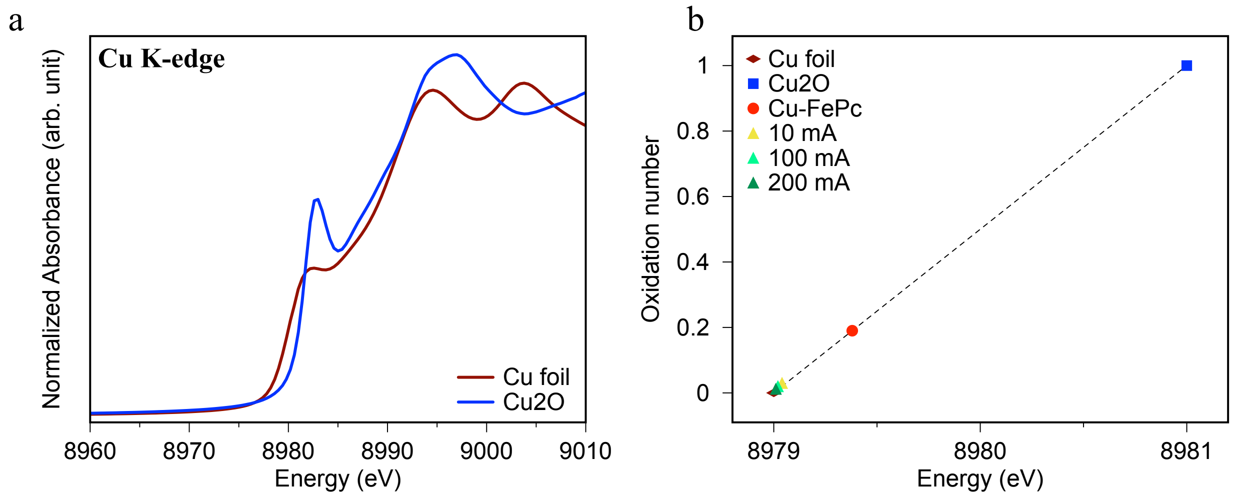

Supplementary Fig. 24 | Identification of oxidation states of Cu operating in flow cell at various current. (**a**) X-ray absorption near edge spectroscopy of standard samples. (**b**) Oxidation states of Cu in flow cell at various current. We use the first inflection point of the spectra as the energy position for identifying the oxidation states.

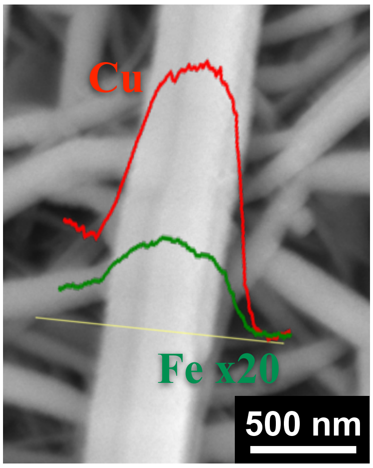

Supplementary Fig. 25 | The elemental analysis via EDX linear scan for single-atom iron on copper.

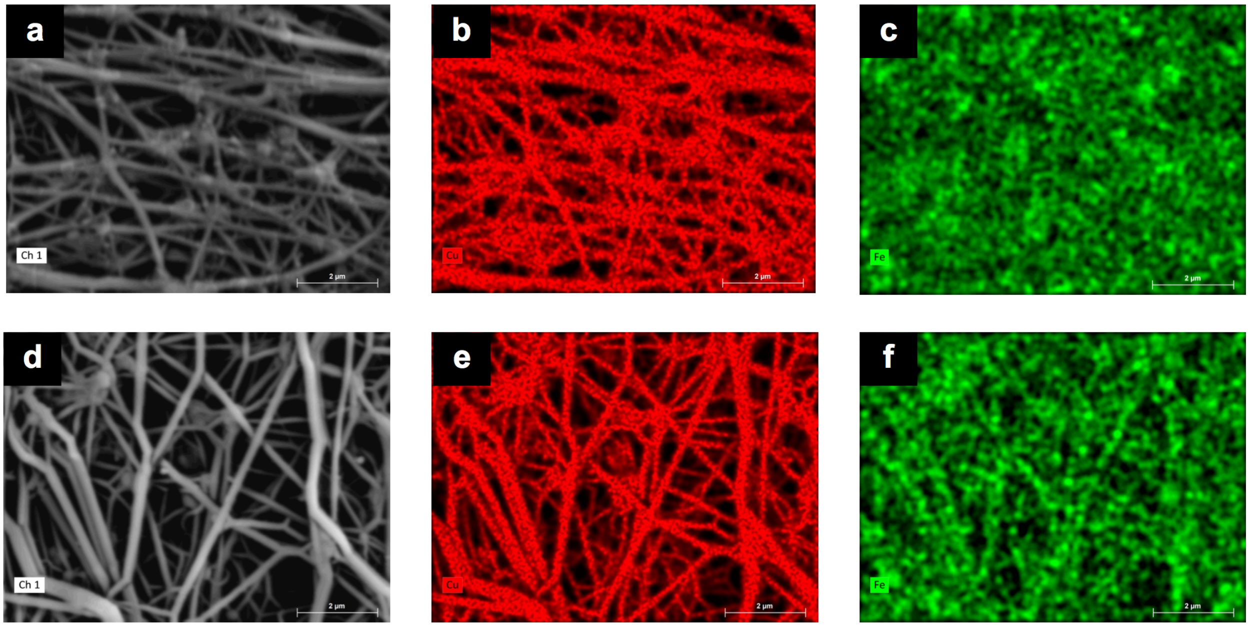

Supplementary Fig. 26 | Elemental analysis of gas diffusion electrode before (a, b, c) and after (d, e, f) catalytic CO_2 reduction reaction. (**a** & **d**) Microstructural images for EDX mapping. (**b** & **e**) EDX mapping of Cu. (**c** & **f**) EDX mapping of Fe.

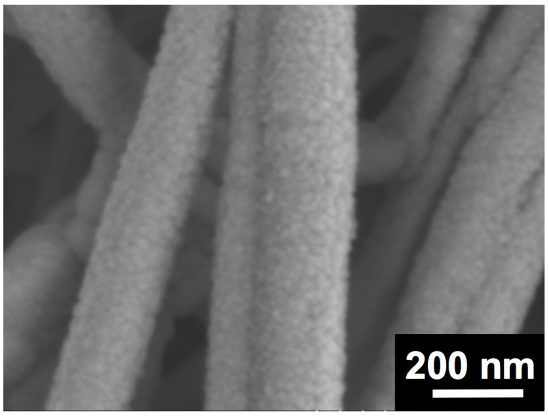

Supplementary Fig. 27 | Microstructure after catalytic CO_2 reduction reaction.

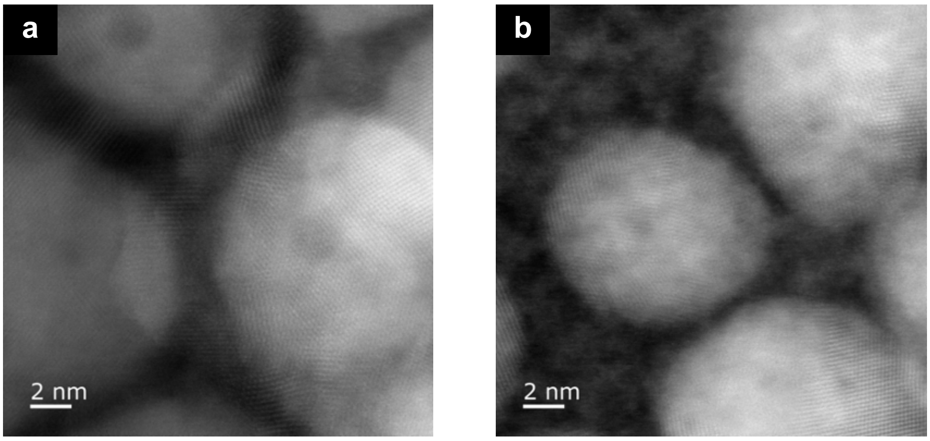

Supplementary Fig. 28 | High-resolution transmission electron microscope images of Cu-FeSA after catalytic CO_2 reduction reaction. (a & b) The HR-TEM images at different places.

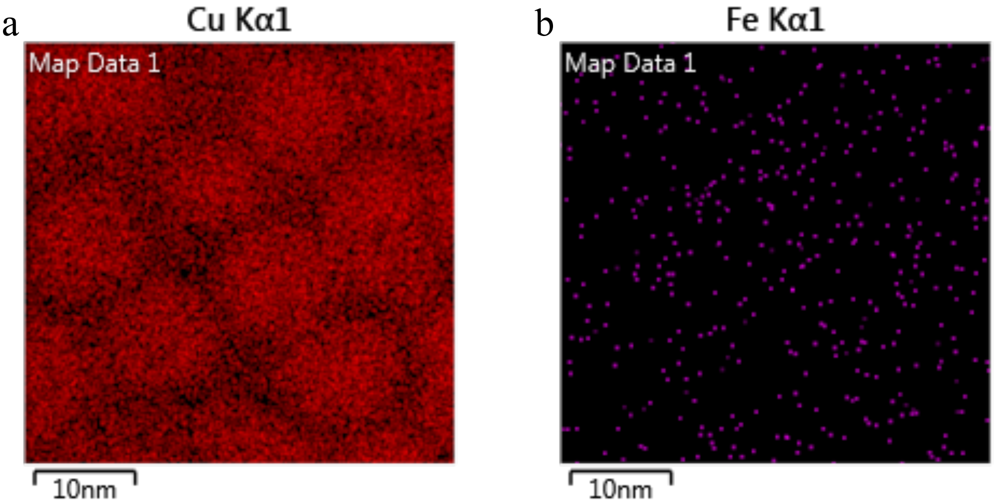

Supplementary Fig. 29 | Elemental analysis of Cu-FeSA after catalytic CO_2 reduction reaction: (a) Cu Kα1 and (b) Fe Kα1. The Fe mapping result shows no aggregated form for iron atoms.

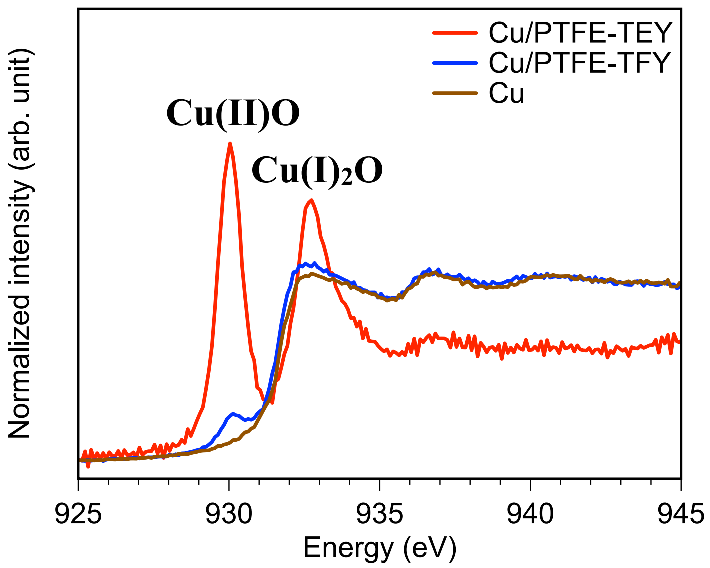

Supplementary Fig. 30 | The Cu L-edge X-ray absorption spectroscopy using total electron yield (TEY, surface sensitive) and total fluorescence yield (TFY, bulk sensitive) to verify the surface oxides on the copper surface.

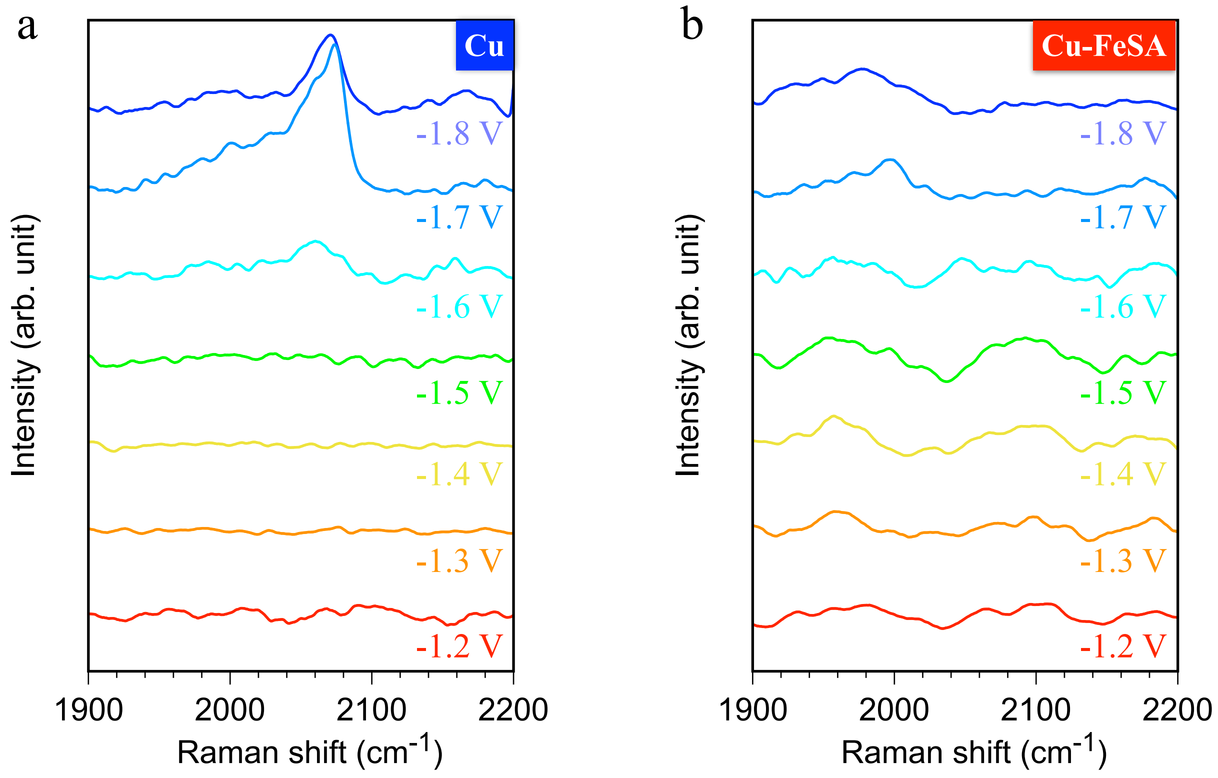

Supplementary Fig. 31 | In-situ Raman spectroscopy for Cu and Cu-FeSA samples. (a) pristine Cu and (b) Cu-FeSA in the expected Raman shift region for the intramolecular C≡O stretch on Cu sites (around 2100 cm^{-1}) and Fe sites (around 1970 cm^{-1}).

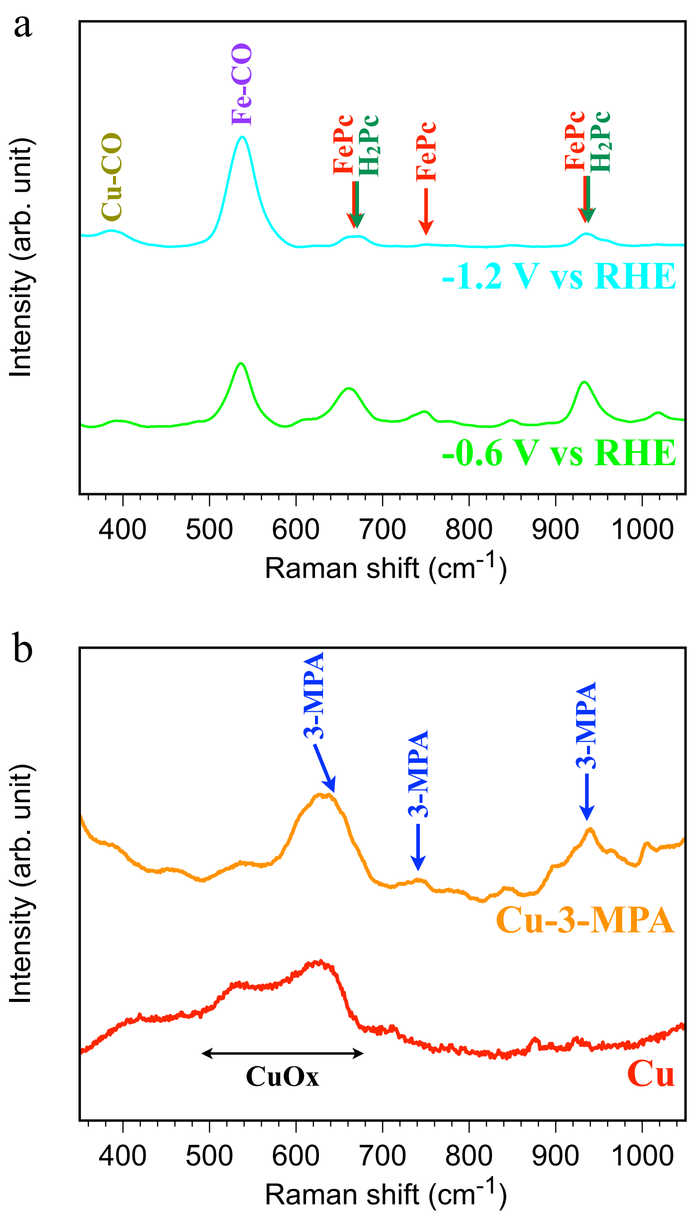

Supplementary Fig. 32 | Comparison of 3-MPA Raman signal between Cu-3-MPA and Cu-FeSA samples. (a) In-situ Raman spectrum of the Cu-FeSA at -0.6 V and -1.2 V vs. RHE. The intensity scale is the same as Figure 3f. (b) Raman spectrum of the as-prepared Cu and Cu-3-MPA samples. The 3-MPA Raman signal vanishes during the catalytic CO_2 reduction reaction, suggesting that 3-MPA leaves the catalytic surface. The signal of H_2Pc remains observable, from which we conclude that iron atoms are attracted to the copper surface, whereas few phthalocyanine rings remain near the copper surface.

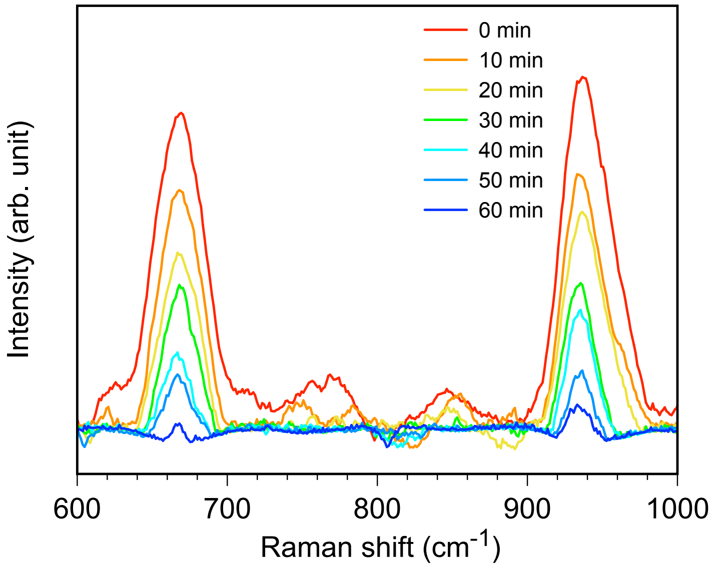

Supplementary Fig. 33 | *In-situ* time-evolution Raman spectrum of Cu-FeSA at -1.2 V vs RHE to identify the remaining phthalocyanine rings.

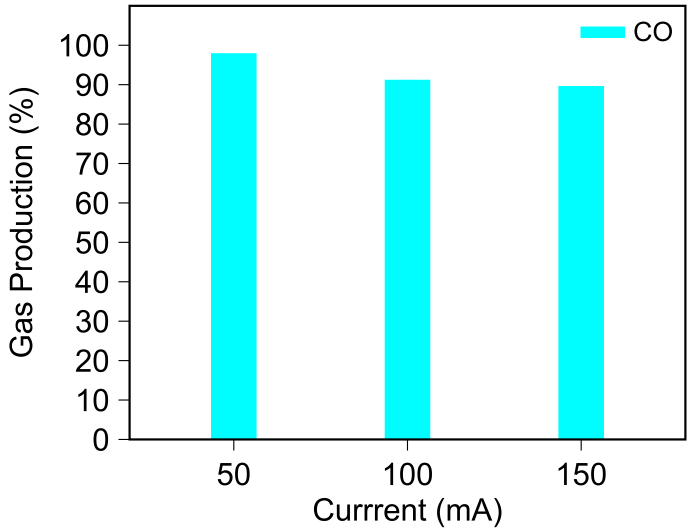

Supplementary Fig. 34 | Catalytic activities of the nitrogen-doped-graphene-supported iron-single-atom at various current.

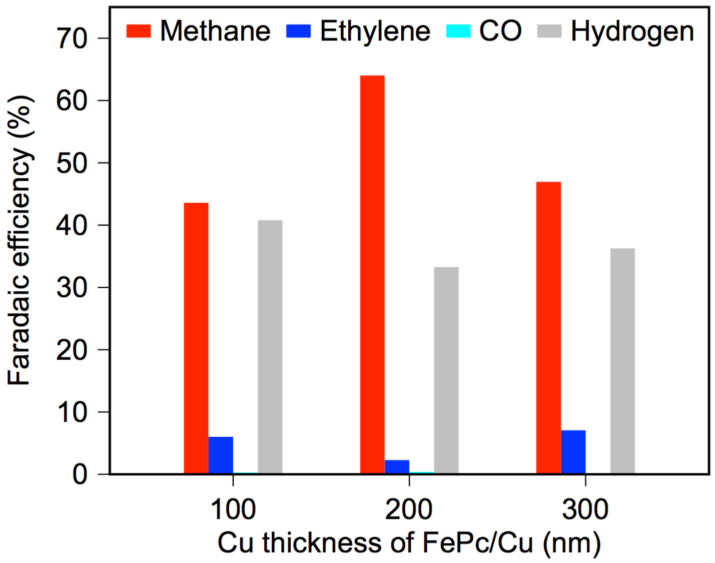

Supplementary Fig. 35 | Catalytic activities of Cu-FeSA at 200 mA/cm^2 with various thicknesses of copper. Three-phase interface is constructed near the boundary between PTFE substrate and catalytic surface. CO_2 can diffuse toward the electrolyte, but the concentration depends on the diffusion distance. The lower thickness of the host copper layer leads to insufficient catalytic sites for CO_2RR, while the increased thickness leads to inadequate CO_2 reaching the catalytic surface. Thus, we obtain an optimal thickness of the host copper layer.

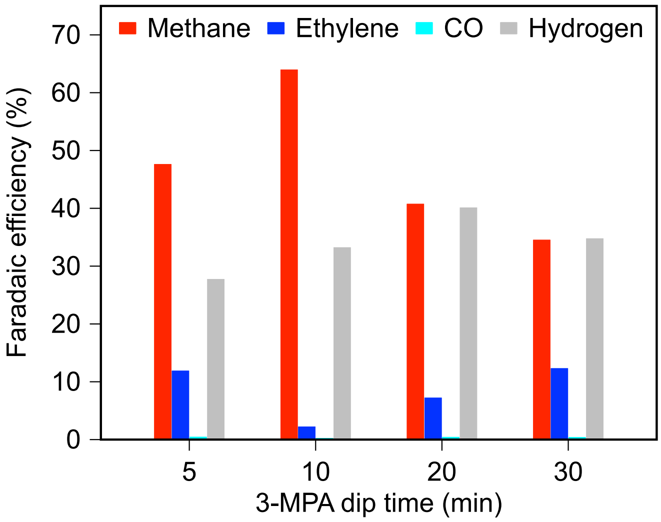

Supplementary Fig. 36 | Catalytic activities of Cu-FeSA at 200 mA cm^{-2} with various 3-MPA dip time. 3-MPA modification time affects the density of the anchored 3-MPA. A lower density of the anchored 3-MPA due to the shorter 3-MPA modification time results in less anchored FePc and fewer single iron atoms. Otherwise, a high density of the anchored 3-MPA will interfere with the bonding process because one of the anchored 3-MPA bonds with the central Fe atom of FePc, but the other anchored 3-MPA nearby cannot bond with the phthalocyanine ring and prevent the access of FePc to the Cu surface.

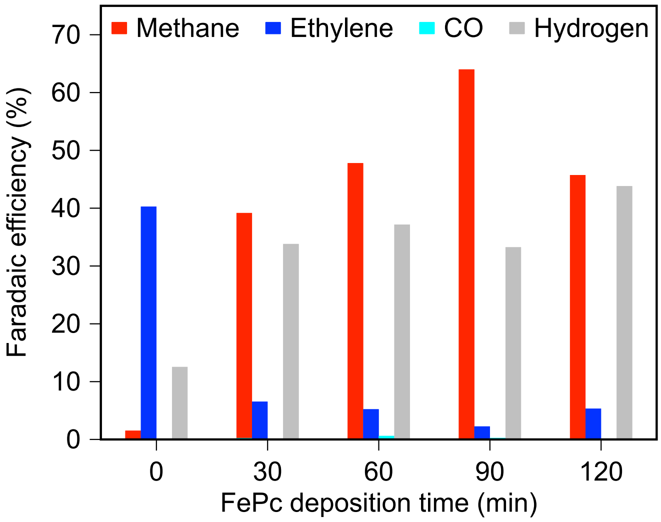

Supplementary Fig. 37 | Catalytic activities of Cu-FeSA at 200 mA/cm^2 with various FePc deposition times. All the samples were treated using 3-MPA surface modification. The slightly decreased ethylene production and the increased hydrogen production of 3-MPA modified copper compared to pristine copper may be related to the fact that 3-MPA alters the copper surface and reduces CO_2RR activity slightly. The Fe compositions for the 30 min, 60 min, 90 min, and 120 min of the FePc deposition time are 0.21 at%, 0.27 at%, 0.30 at%, and 0.35 at%, using inductively coupled plasma mass spectrometry (ICP-MS). We notice that the initial adsorption of FePc is a rapid process but slows down after the longer deposition time.

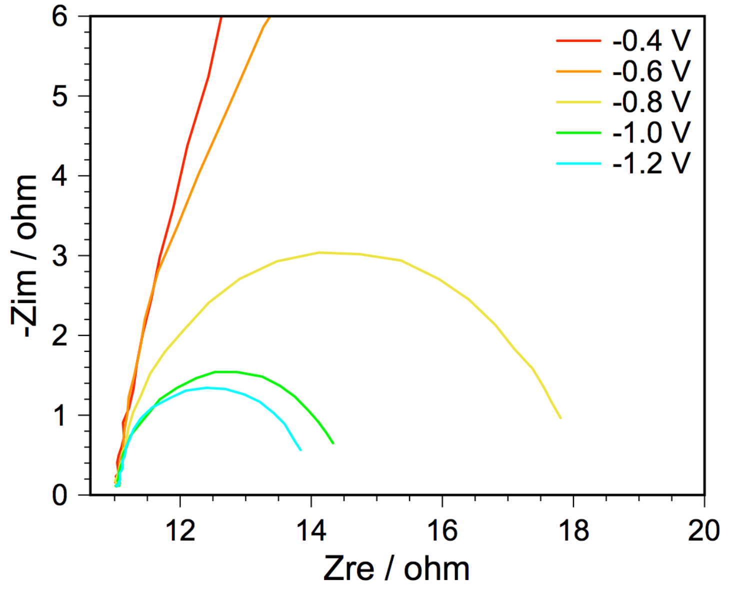

Supplementary Fig. 38 | Nyquist plots of Cu-FeSA recorded at various applied voltages, which are referred to RHE, for CO_2RR in flow cell.

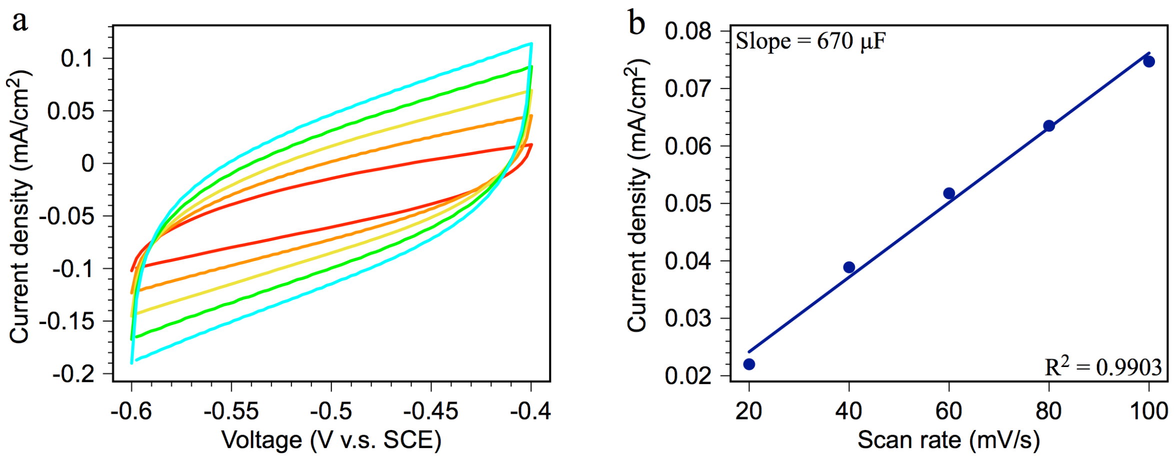

Supplementary Fig. 39 | Cyclic voltammogram for electrochemical active surface area. (a) Cyclic voltammograms in the non-faradaic region at scan rate of 20 mV/s (red), 40 mV/s (orange), 60 mV/s (yellow), 80 mV/s (green), and 100 mV/s (cyan) for Cu-FeSA (b) Capacitive current density as a function of scan rate. The slope is the electrochemical double-layer capacitance, which is proportional to the morphological surface area or roughness factor.

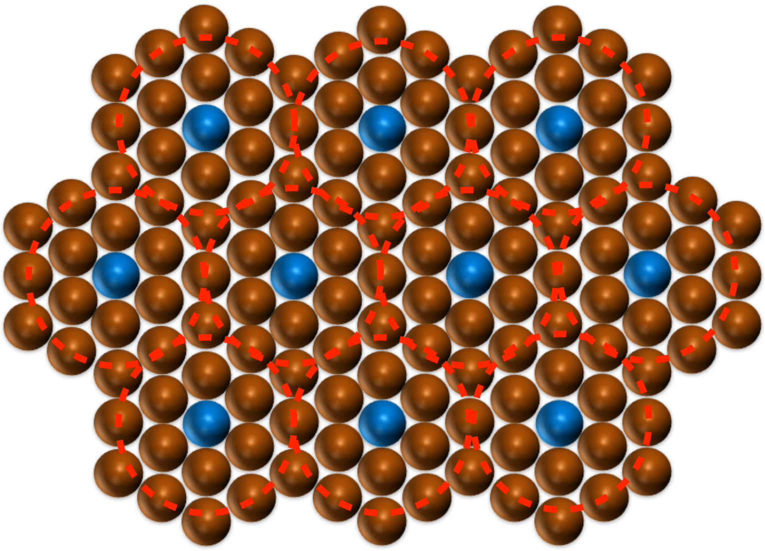

Supplementary Fig. 40 | The uniform atomic arrangement in the atomic ratio of Cu:Fe (14:1). The red circles imply the migration region of CO intermediates toward Fe atom, obtained by the results in Supplementary Fig. 4.

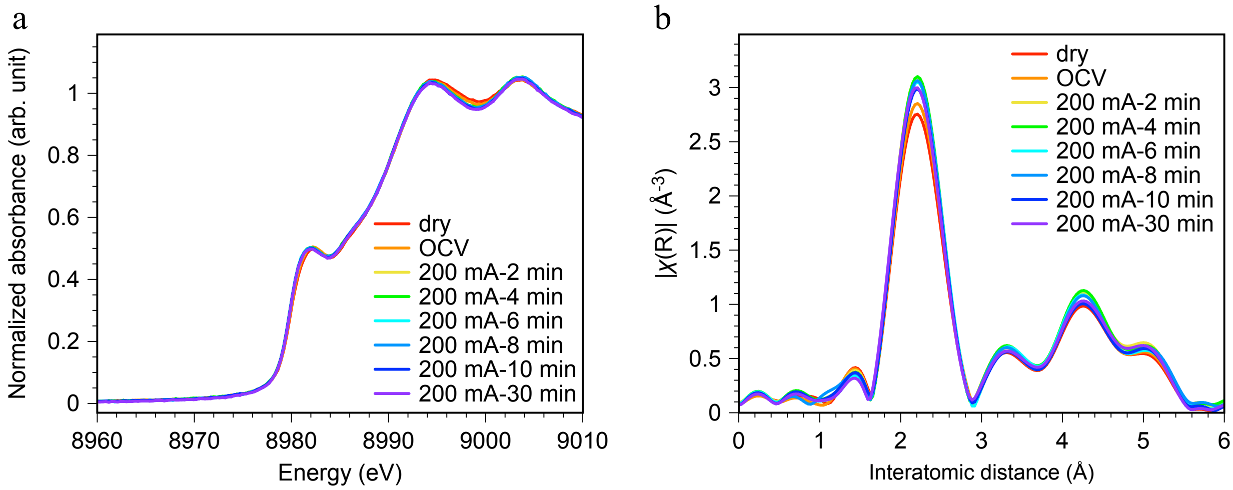

Supplementary Fig. 41 | In-situ time-resolved Cu K-edge X-ray absorption spectroscopy operating in flow cell at 200 mA cm^{-2}. (**a**) In-situ time-resolved X-ray absorption near-edge structure (XANES) for Cu K-edge. (**b**) In-situ time-resolved extended X-ray absorption fine structure (EXAFS) for Cu K-edge. OCV means open circuit voltage.

Supplementary Table 1. Surface energy results of low-index facets of Cu from DFT calculations

Cu facets	Surface energies (eV/ Å^2)
(100)	0.076
(110)	0.087
(111)	0.066

Supplementary Table 2. Comparison of electrochemical CO_2-to-methane performance with total current density higher than 100 mA cm^{-2}.

Catalyst	FE (%)	$J_{methane}$ (mA/cm^2)	J_{total} (mA/cm^2)	Reference
Cu-FeSA	64	128	200	This work
Cu	48	108	225	*J. Am. Chem. Soc.* 2020, 142, 3525
Cu-complex	1	3	300	*Nat. Catal.* 2020, 3, 75
Cu-molecular	0.5	1.61	322	*Nature* 2020, 577, 509
Cu	18	19	106	*Nat. Catal.* 2018, 1, 111
ERD Cu	0.18	0.81	450	*Nat. Catal.* 2018, 1, 103

References:

1. Chase, M. W. NIST-JANAF thermochemical tables for oxygen fluorides. *J. Phys. Chem. Ref. data* **25**, 551-603 (1996).
2. Rossmeisl, J., Qu, Z.-W., Zhu, H., Kroes, G.-J., Nørskov, J. K. Electrolysis of water on oxide surfaces, *J. Electroanal. Chem.* **607**, 83-89 (2007).
3. Nørskov, J. K., Rossmeisl, J., Logadottir, A., Lindqvist, L., Kitchin, J. R., Bligaard, T., Jónsson, H. Origin of the overpotential for oxygen reduction at a fuel-cell cathode. *J. Phys. Chem. B* ***108***, 17886-17892 (2004).
